# Association Between SGLT2 Inhibitor Therapy and the Incidence of Tinnitus in Patients with Type 2 Diabetes: A Retrospective Cohort Study

**DOI:** 10.3390/audiolres15040102

**Published:** 2025-08-09

**Authors:** David Ulrich Seidel, Simon Bode, Karel Kostev

**Affiliations:** 1 Department of Otorhinolaryngology, Facial Plastic Surgery, Klinikum Oberberg, 51643 Gummersbach, Germany; 2 University Hospital, Philipps University, 35043 Marburg, Germany; 3 Epidemiology, IQVIA, 60549 Frankfurt am Main, Germany

**Keywords:** tinnitus, diabetes, SGLT2 inhibitor, sodium-glucose cotransporter-2 inhibitor, practice database, Germany

## Abstract

Background: Numerous studies have demonstrated the beneficial effects of sodium-glucose cotransporter-2 (SGLT2) inhibitors on cardiovascular and renal outcomes in patients with heart failure and chronic kidney disease. However, whether SGLT2 inhibitors are also associated with a reduced risk of tinnitus has not been investigated. Objective: This study aimed to investigate the association between SGLT2 inhibitor therapy and the incidence of tinnitus in patients with type 2 diabetes. Methods: This retrospective cohort study was based on data from a nationally representative database of primary care practices in Germany from 2012 to 2023. Patients with type 2 diabetes who were treated with metformin and additionally received either an SGLT2 inhibitor or a dipeptidyl peptidase-4 (DPP4) inhibitor were included. Patients with a previous diagnosis of tinnitus were excluded. The primary outcome was the first tinnitus diagnosis documented by a primary care physician. The SGLT2 and DPP4 cohorts were compared for tinnitus incidence using Kaplan–Meier analysis and multivariable Cox regression. Results: 66,750 patients with SGLT2 inhibitors and 82,830 with DPP4 inhibitors were analyzed. The cumulative 5-year incidence of tinnitus was 1.9% in both groups. The multivariable regression analysis did not show a significant association between SGLT2 therapy and the occurrence of a tinnitus diagnosis (HR: 1.04; 95% CI: 0.89–1.21). Conclusion: There was no difference in tinnitus incidence between patients with SGLT2 or DPP4 inhibitors. The causes could lie in the heterogeneous, not purely vascular, etiology of tinnitus in general practitioners’ practices. Future studies should include further clinical data, including confirmed hearing impairments.

## 1. Introduction

Tinnitus is one of the most common otologic symptoms, affecting approximately 14% to 37% of the population [1]. It is defined by the perception of sound in the absence of an external acoustic stimulus. The etiology is diverse and includes not only otologic but also macrovascular (e.g., pulsatile tinnitus), neurological, and psychogenic factors. The most frequent pathophysiological correlate of tonal tinnitus is inner ear damage in the form of sensorineural hearing loss. Microvascular alterations are thought to contribute to the development of both hearing loss and tinnitus [2,3,4]. Numerous studies have identified codiagnoses that are frequently associated with tinnitus, including arterial hypertension, atherosclerosis, dyslipidemia, various forms of hearing loss and other inner ear disorders, as well as depression [5,6,7,8,9].

Type 2 diabetes mellitus is a common chronic metabolic disorder that is associated with an increased risk of cardiovascular complications. Several studies have suggested a link between diabetes and auditory disorders, including tinnitus. Possible mechanisms include auditory neuropathy, oxidative stress, and reduced blood flow to the inner ear due to diabetes-related microangiopathy, which may contribute to the development of tinnitus [10,11]

In recent years, an increasing number of studies have highlighted the additional health benefits of modern antidiabetic agents—particularly sodium-glucose cotransporter-2 (SGLT2) inhibitors. Beyond their glucose-lowering effects, SGLT2 inhibitors have been shown to reduce the risk of major cardiovascular events and the progression of chronic kidney disease [12,13,14]. Moreover, some studies have reported associations with a lower risk of stroke, dementia, and anemia [14,15,16,17]. SGLT inhibitors act by inhibiting glucose and sodium reabsorption in the proximal renal tubules, thereby promoting glucosuria and lowering blood glucose levels. Beyond their glucose-lowering effects, several additional mechanisms are currently under investigation. These include the modulation of the renin–angiotensin–aldosterone system (RAAS), the improvement of vascular endothelial function through anti-inflammatory and antifibrotic effects, and a reduction in vascular tone [15].

As mentioned above, numerous studies have demonstrated significant associations between hearing loss, tinnitus, and cardiovascular risk factors and diseases. The underlying mechanisms are thought to involve endothelial dysfunction, atherosclerosis, and impaired microcirculation of the inner ear [2,3,5,6,7]. This raises the question of whether SGLT2 inhibitors are associated not only with a reduced risk of cardiovascular disease, but also with a reduced risk of inner ear disorders such as sensorineural hearing loss or tinnitus.

The aim of the present study was to investigate this potential association using data from general practitioners documented in a nationally representative practice database.

## 2. Material and Methods

### 2.1. Data Source

This analysis is based on data from the IQVIA™ Disease Analyzer database, which contains patient-level information from approximately 3500 general practitioners and specialists in private practice in Germany. The database contains records on demographic characteristics, diagnoses, drug prescriptions, and laboratory values, covering more than eight million patients between 2012 and 2023. The included practices are geographically representative of Germany, covering eight major regions. The sampling methods used to select participating practices have been shown to be appropriate for generating a representative dataset for outpatient general and specialist care in Germany [16].

**Figure 1:**
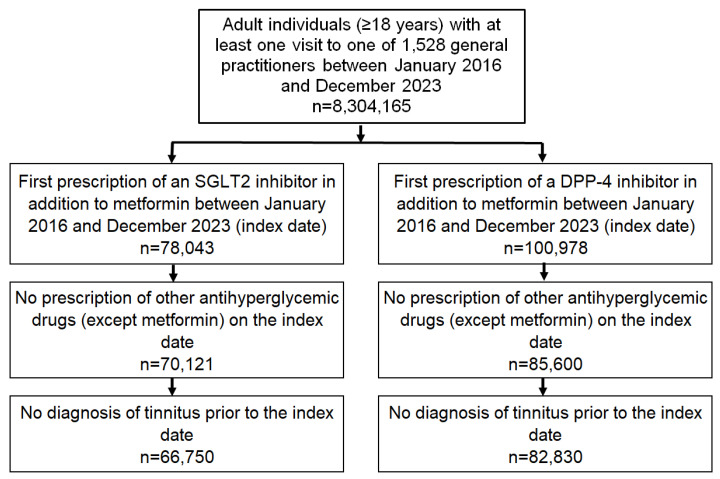
Selection of study patients.

This retrospective cohort study included patients with a confirmed diagnosis of type 2 diabetes (ICD-10: E11) who were prescribed either a sodium-glucose cotransporter-2 (SGLT2) inhibitor (ATC: A10BK) or a dipeptidyl peptidase-4 (DPP4) inhibitor (ATC: A10BH) in addition to metformin (ATC: A10BA) between January 2016 and December 2023 (index date). If both classes of agents were prescribed during the study period, the agent prescribed first was selected. Patients were excluded if they had received prescriptions for other glucose-lowering agents (except metformin) on the index date or had a documented diagnosis of tinnitus (ICD-10: H93.1) before or on the index date in order to reduce potential confounding by pre-existing tinnitus (Figure 1).

Patients were divided into two cohorts: one receiving SGLT2 inhibitors and the other receiving DPP4 inhibitors. The two groups were compared for the incidence of tinnitus. The following covariates were recorded: age, sex, year of treatment initiation (2012–2023), and comorbidities documented within the 12 months prior to the index date that have been associated with tinnitus in previous studies. These included vestibular dysfunction (ICD-10: H81), various forms of hearing loss (ICD-10: H90, H91), arterial hypertension (ICD-10: I10), atherosclerosis (ICD-10: I70), dyslipidemia (ICD-10: E78), chronic obstructive pulmonary disease (COPD, ICD-10: J44), and depression (ICD-10: F32) [5,6,7,8,9]. In addition, the duration of diabetes and baseline HbA1c were assessed.

### 2.2. Statistical Analyses

Differences in baseline characteristics between the SGLT2 and DPP4 cohorts were analyzed using the *t*-test (for mean age), the Wilcoxon signed-rank test (for median diabetes duration), and the Chi^2^ test (for categorical variables).

The primary endpoint was the cumulative incidence of tinnitus diagnoses under SGLT2 or DPP4 therapy. Patients were followed for a maximum of five years starting from the index date. Follow-up ended at the earliest occurrence of one of the following events: (1) a first diagnosis of tinnitus, (2) a change in antidiabetic therapy (i.e., initiation of any additional glucose-lowering drug), (3) loss to follow-up, or (4) the end of the observation period (31 December 2023). This ensured a consistent observation window while accounting for treatment changes and data completeness.

The Kaplan–Meier method was used to estimate the cumulative incidence of tinnitus. Multivariable Cox regression models were used to examine the association between treatment type and tinnitus risk. These were adjusted for age, sex, year of treatment initiation, duration of diabetes, relevant comorbidities, and baseline HbA1c. Additional stratified analyses were performed by age group, sex, and year of treatment initiation. As sensitivity analyses, multivariable regression analyses were conducted for patients with hypertension, atherosclerosis, and ischemic heart diseases (ICD-10: I20–I25). A *p*-value < 0.05 was considered statistically significant. All analyses were performed with SAS version 9.4 (SAS Institute, Cary, NC, USA).

## 3. Results

Baseline Characteristics of the Study Population

A total of 66,750 patients receiving SGLT2 inhibitor therapy and 82,830 patients receiving DPP4 inhibitor therapy were included in the analysis. Table 1 shows the baseline characteristics of both cohorts. The median age was 68 years in both groups. The proportion of female patients was higher in the SGLT2 cohort (45.7% vs. 38.6%).

The prevalence of all comorbidities was slightly higher in the SGLT2 group. The largest difference was observed in the year of therapy initiation: the majority of SGLT2 prescriptions were made in 2022 and 2023, whereas DPP4 inhibitors were mainly prescribed before 2022 (see Table 1). No relevant differences were found in baseline HbA1c levels (7.7–7.9%) or in the median diabetes duration at the index date (1 year).

**Figure 2:**
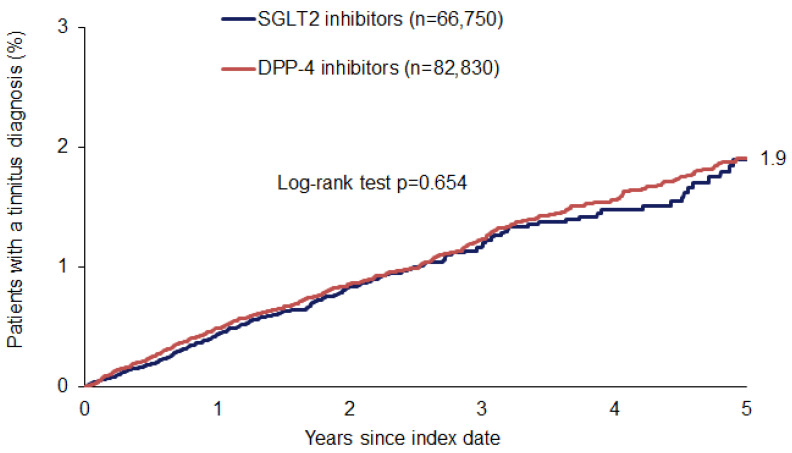
Cumulative incidence of tinnitus diagnoses in patients with type 2 diabetes treated with SGLT2 or DPP4 inhibitors (Kaplan–Meier curves).

The cumulative 5-year incidence of tinnitus diagnoses was 1.9% in both cohorts (Figure 2). The results of the multivariable Cox regression analysis are shown in Table 2. No significant association was observed between SGLT2 therapy and the incidence of tinnitus (hazard ratio: 1.04; 95% confidence interval: 0.89–1.21).

Stratified analyses by age group, sex, and year of therapy initiation also showed no statistically significant associations with tinnitus diagnoses (Table 2). Furthermore, no significant associations between SGLT2 therapy and tinnitus were observed in patients with co-diagnoses of hypertension, atherosclerosis, and ischemic heart diseases.

## 4. Discussion

This retrospective cohort study examined the association between the use of SGLT2 inhibitors and the occurrence of tinnitus in patients with type 2 diabetes using primary care data from a nationally representative practice database. Multivariable regression analyses revealed no significant difference in the incidence of tinnitus between patients treated with SGLT2 inhibitors and those treated with DPP4 inhibitors. To the authors’ knowledge, this is the first study to examine the association between SGLT2 inhibitor therapy and tinnitus.

### 4.1. Considerations Regarding the Study Design

The aim of this study was to investigate a possible association between the use of SGLT2 inhibitors and inner ear dysfunction, primarily in the form of sensorineural hearing loss, which may in part be related to microvascular damage. Given the proposed mechanisms of SGLT2 inhibitors beyond glycemic control—such as blood pressure reduction, anti-inflammatory and antifibrotic effects, the improvement of endothelial function, and reductions in vascular tone [15]—it is conceivable that microvascular alterations associated with sensorineural hearing loss and tinnitus may be positively influenced by these agents. However, because such hearing loss is rarely diagnosed by general practitioners and is instead typically identified by ENT specialists, whose data do not include relevant internal comorbidities, medication use, or laboratory parameters, a direct association between hearing impairment and antidiabetic therapy could not be established. In contrast, tinnitus diagnoses are regularly documented by general practitioners. Therefore, tinnitus was selected as a pragmatic study endpoint to allow the investigation of potential associations between SGLT2 inhibitor use and inner ear involvement based on routine data from general practices.

An evaluation of the practice database during the study design phase showed that, after initial metformin monotherapy, SGLT2 and DPP4 inhibitors are currently the most commonly prescribed second-line antidiabetic agents by general practitioners in Germany. The comparison of these two drug classes therefore reflects their current relevance in general practice. Other antidiabetic agents, such as sulfonylureas or GLP-1 receptor agonists, are less commonly prescribed and would have a reduced statistical power due to smaller case numbers.

For this reason, the selection of these two classes of drugs seemed appropriate in order to establish a comparison group with the highest possible degree of homogeneity. The aim was to compare two cohorts of patients with similar baseline characteristics to allow for an unbiased analysis of the association between each medication and the occurrence of tinnitus. It is important to note that SGLT2 inhibitors exhibit cardiovascular protective effects, whereas DPP4 inhibitors do not—making these two drug classes a suitable comparison for evaluating potential vascular influences [13].

### 4.2. Tinnitus Incidence and Demografic Data

The observed 5-year incidence of tinnitus in this primary care cohort is comparatively low. A recent meta-analysis estimated a global annual incidence of around 1% [1]. It is therefore plausible that many patients with newly onset tinnitus sought care directly from ENT specialists rather than from their general practitioners. As a result, not all tinnitus cases may have been captured in the primary care database, and systematic underreporting cannot be ruled out.

The age distribution was similar in both cohorts. The SGLT2 group had a slightly higher proportion of female patients. A notable difference was observed in the year of therapy initiation: while DPP4 inhibitors were predominantly prescribed between 2016 and 2021, prescriptions of SGLT2 inhibitors increased significantly, especially from 2022 onward.

This shift likely reflects the growing body of evidence supporting the use of SGLT2 inhibitors. A recent systematic review and network meta-analysis commissioned by the American College of Physicians showed that, compared with DPP4 inhibitors, SGLT2 inhibitors significantly reduced all-cause mortality, the risk of cardiovascular events, the progression of chronic kidney disease, and hospitalizations for heart failure [13].

### 4.3. Possible Confounders

The comorbidities previously identified as being associated with tinnitus were considered as potential confounders in this study. These included vestibular dysfunction, hearing loss, hypertension, atherosclerosis, dyslipidemia, chronic obstructive pulmonary disease (COPD), and depression [5,6,7,8,9]. These diagnoses were slightly more common in the SGLT2 cohort. This may be due to selective prescribing patterns, as current evidence suggests that SGLT2 inhibitors are preferentially prescribed to patients with pre-existing cardiovascular disease.

The higher prevalence of depression and hearing loss in this group may be explained by known associations with cardiovascular disease [2,17]. In contrast, a potential association between vestibular dysfunction and cardiovascular disease has not been systematically investigated in large studies. Some smaller studies have shown inconsistent results [18,19,20,21]. The slightly increased prevalence of vestibular dysfunction in the SGLT2 group may be an indirect indicator of a possible association with cardiovascular risk.

The mean duration of diabetes and baseline HbA1c levels were nearly identical in both cohorts, which is essential to ensure comparability between the groups.

A 1:1 matching of the cohorts was not performed, as this would have resulted in a substantial reduction in sample size and, consequently, a loss of statistical power. Instead, potential confounders were adjusted for in multivariable regression analyses. This approach allowed statistical control for group differences and allowed the evaluation of the independent association between each antidiabetic therapy and the incidence of tinnitus diagnoses.

### 4.4. Interpretation of the Results

SGLT2 inhibitors have been shown in prospective studies to be protective against heart failure and renal impairment and are therefore increasingly prescribed to patients with these comorbidities. In addition, retrospective studies have reported associations with a reduced risk of other conditions such as stroke, dementia, and anemia [12,13,14,22,23,24]. In this study, however, no association was observed between the use of SGLT2 inhibitors and a lower incidence of tinnitus, either overall or in age-, sex-, or time-specific subgroups. The frequency of newly diagnosed tinnitus cases was identical in both cohorts.

One possible explanation for this finding is the heterogeneity of tinnitus diagnoses in general practice. It remains unclear to what extent these diagnoses actually reflect tinnitus of inner ear origin, which may be a vascular disease, or whether they represent other forms, such as those associated with middle ear pathology, Ménière’s disease, macrovascular malformations with pulsatile tinnitus, retrocochlear disorders, or psychogenic causes.

In many cases, patients consult their general practitioner primarily because of the subjective distress caused by tinnitus—regardless of its underlying etiology. These encounters typically do not involve otologic diagnostics, but rather focus on the psychosocial impact of the symptom, which may lead to further clinical consequences such as the initiation of antidepressant therapy. As a result, the recorded diagnoses may reflect perceived suffering more than a clearly defined auditory pathology.

Additional explanations for the absence of an observed association include a limited follow-up duration in some patients and a generally low incidence of tinnitus in this population, which may have reduced the statistical power to detect a potential effect. Investigating the association between SGLT2 therapy and sensorineural hearing loss diagnosed by ENT specialists could offer further insights.

### 4.5. Strengths and Limitations of the Study

This study has several limitations that should be considered when interpreting the results. The analysis is based solely on diagnoses documented by general practitioners using ICD-10 codes, while detailed clinical information is not available in the database. For example, no audiological findings that would allow differentiation between different types of tinnitus were recorded. Therefore, it remains unclear to what extent documented tinnitus diagnoses were related to vascular factors or to other causes such as middle ear disease, Ménière’s disease, macrovascular malformations, retrocochlear pathology, or psychological distress. As a result, the robustness of the study endpoint is limited.

Moreover, it is possible that tinnitus diagnoses were systematically underreported in this primary care sample, as patients may have sought care directly from ENT specialists. Consequently, not all cases of tinnitus may have been captured, which could limit the representativeness of the findings.

In addition, other potential confounding factors such as noise exposure, smoking status, physical activity, or stress levels are not systematically recorded. Due to the retrospective design of the study, only associations can be examined, and no causal conclusions can be drawn.

Despite these limitations, the study has several notable strengths. These include the use of a nationally representative primary care database and the large sample size, which allows for statistically robust analyses. The continuous documentation of diagnoses, prescriptions, and laboratory values as part of routine primary care reduces the risk of recall bias. The inclusion of HbA1c levels and diabetes duration accounted for two important clinical variables. In addition, the comparison of the two most commonly prescribed oral second-line antidiabetic drug classes—SGLT2 inhibitors and DPP4 inhibitors—provided a relatively homogeneous comparison group, thereby strengthening the validity of the results.

## 5. Conclusions

Previous prospective studies have demonstrated the benefits of SGLT2 inhibitors over other antidiabetic agents in terms of cardiovascular outcomes. Since tinnitus may, at least in part, be linked to vascular mechanisms, the potential influence of SGLT2 inhibitors on tinnitus risk has been hypothesized. However, the present retrospective analysis found no association between SGLT2 therapy and a reduced incidence of tinnitus. Future studies that integrate data from both general practitioners and ENT specialists could provide a more comprehensive understanding of inner ear disorders and their potential relationship with antidiabetic therapies.

## Figures and Tables

**Table 1:** Basic characteristics of study patients.

Variable	SGLT2 Patients	DPP4 Patients	*p* Values
Number of patients	66,750	82,830	
Age mean (SD)	68.4 (13.3)	68.1 (13.0)	<0.001
≤60 years	18,515 (27.7)	23,469 (28.4)	<0.001
61–70 years	17,388 (26.1)	21,546 (26.0)	
71–80 years	16,305 (24.4)	21,480 (25.9)	
>80 years	14,542 (21.8)	16,335 (19.7)	
Sex: female	25,749 (45.7)	37,837 (38.6)	<0.001
Sex: male	41,001 (54.3)	44,993 (61.4)	
Start of therapy: 2016–2018	8164 (12.2)	33,317 (40.2)	<0.001
Start of therapy: 2019–2021	18,486 (27.7)	32,840 (39.7)	
Start of therapy: 2022–2023	40,100 (60.1)	16,673 (20.1)	
Disorders of vestibular function	2359 (3.5)	2025 (2.4)	<0.001
All-cause hearing loss	3728 (5.6)	3438 (4.2)	<0.001
Hypertension	44,916 (67.3)	49,097 (59.3)	<0.001
Atherosclerosis	5453 (8.2)	4394 (5.3)	<0.001
Dyslipidemia	29,609 (44.4)	29,584 (35.7)	<0.001
COPD	9500 (14.2)	8403 (10.1)	<0.001
Depression	13,346 (20.0)	13,800 (16.7)	<0.001
HbA1c in % mean (SD)	7.7 (1.7)	7.9 (1.6)	<0.001
Diabetes duration median (IQR)	1.0 (5.2)	1.0 (5.2)	<0.001

Values are given as absolute numbers and percentages, unless indicated otherwise. SD: standard deviation.

**Table 2:** Association between SGLT2 therapy and the risk of tinnitus (age- and sex- stratified multivariable Cox regression analyses *).

Subgroup	Adjusted HR (95% CI) *	*p* Value
Total cohort	1.04 (0.89–1.21)	0.641
Patients aged ≤60 years	1.09 (0.86–1.39)	0.479
Patients 61–70 years	1.05 (0.80–1.38)	0.732
Patients 71–80 years	1.00 (0.69.–1.46)	0.996
Patients >80 years	0.95 (0.58–1.58)	0.086
Female patients	1.24 (0.97–1.59)	0.087
Male patients	0.93 (0.76–1.13)	0.439
Start of therapy: 2016–2018	1.22 (0.95–1.58)	0.125
Start of therapy: 2019–2021	0.89 (0.70–1.14)	0359
Start of therapy: 2022–2023	0.99 (0.72–1.37)	0.971
Patients with hypertension	0.99 (0.82–1.20)	0.936
Patients with atherosclerosis	1.01 (0.54–1.90)	0.969
Patients with ischemic heart disease	1.16 (0.85–1.59)	0.356

* adjusted for age, sex, year of therapy initiation, diabetes duration, co-diagnoses, and HbA1c value.

## Data Availability

The data and the code used for this study are available from the corresponding author upon request.
